# Tracking and Recognition of Multiple Human Targets Moving in a Wireless Pyroelectric Infrared Sensor Network

**DOI:** 10.3390/s140407209

**Published:** 2014-04-22

**Authors:** Ji Xiong, Fangmin Li, Ning Zhao, Na Jiang

**Affiliations:** Key Laboratory of Fiber Optic Sensing Technology and Information Processing, School of Information and Engineering, Wuhan University of Technology, Wuhan 430070, Hubei, China; E-Mails: bear-xiongji@163.com (J.X.); zhaoning@whut.edu.cn (N.Z.); WHUTjiangna@163.com (N.J.)

**Keywords:** multiple human identification, distributed wireless pyroelectric sensor network, empirical mode decomposition, feature extracting

## Abstract

With characteristics of low-cost and easy deployment, the distributed wireless pyroelectric infrared sensor network has attracted extensive interest, which aims to make it an alternate infrared video sensor in thermal biometric applications for tracking and identifying human targets. In these applications, effectively processing signals collected from sensors and extracting the features of different human targets has become crucial. This paper proposes the application of empirical mode decomposition and the Hilbert-Huang transform to extract features of moving human targets both in the time domain and the frequency domain. Moreover, the support vector machine is selected as the classifier. The experimental results demonstrate that by using this method the identification rates of multiple moving human targets are around 90%.

## 1. Introduction

The development of modern science and technology has made people's living environments gradually become intelligent and networked. Meanwhile, all sorts of environmental monitoring systems [1,2], security systems and intelligent auxiliary systems [3] are increasingly being widely used in the home, office, factory or harsh working conditions such as mines. In these applications, the accurate identification of moving human targets is crucial for physical protection and emergency relief, *etc.* Generally, systems for the tracking and recognition of human targets [4] need to collect stable and reliable physical characteristics [5] such as fingerprints, iris, voice spectra, facial features, or behavioral characteristics such as signatures and gait in order to realize individual identification and action tracking based on those characteristics. Traditional video systems [6,7] have been applied in many recognition scenarios, however, they mainly identify a person using facial features which are greatly affected by many external factors such as lighting, angle or clothes. In addition, they usually have high computational overhead and require huge data throughput. Recently, it was found that the behavioral characteristics of a human target are independent physical appearances and have strong advantages, compared with other biological characteristics, in an ever-changing environment [8]. In a distant or crowded scene, however, it is a very complicated problem to identify a human target using some behavioral biometric features, whose main reason is closely related to the perception of human object form and feature selection.

In recent years, with the development of the sensor technology, wireless network communication technology, distributed inference and learning technology, signal processing technology, the tracking and recognition of humans using distributed systems have gradually drawn the attention of researchers [9,10]. The behavioral information of a human object can usually be measured by passive or active sensors, and some tiny, cheap, spatially distributed sensors nodes with computation and communication capabilities can work together to complete complex tasks. Therefore, researchers have attempted to combine their virtues and develop distributed sensor networks based on pyroelectric infrared sensor nodes in order to realize tracking and recognition of human objects [4,10]. The pyroelectric infrared sensor has various advantages [9,11,12]: (1) high capability in detecting infrared radiation; (2) low power consumption (about 2 mW) and a small amount of calculation; (3) its performance is independent of illumination and has strong robustness to color changes of the background; (4) its angular rate sensitivity range is about 0.1 r/s to 3 r/s, which can cover most human walking speeds of t around 2–10 m; (5) it can only respond to a moving target; (6) it can obtain a better field of view (FOV) combined with low price Fresnel lens arrays. Thus, compared with the traditional video system, using a distributed wireless pyroelectric sensor network can provide better spatial coverage and reduce the time and place restrictions of system deployment. Meanwhile, the characteristics of the pyroelectric infrared sensor make the system have the ability to be operated under any illumination condition, as well as the ability to capture the statistical characteristics of the human body temperature [13].

**Figure 1.**
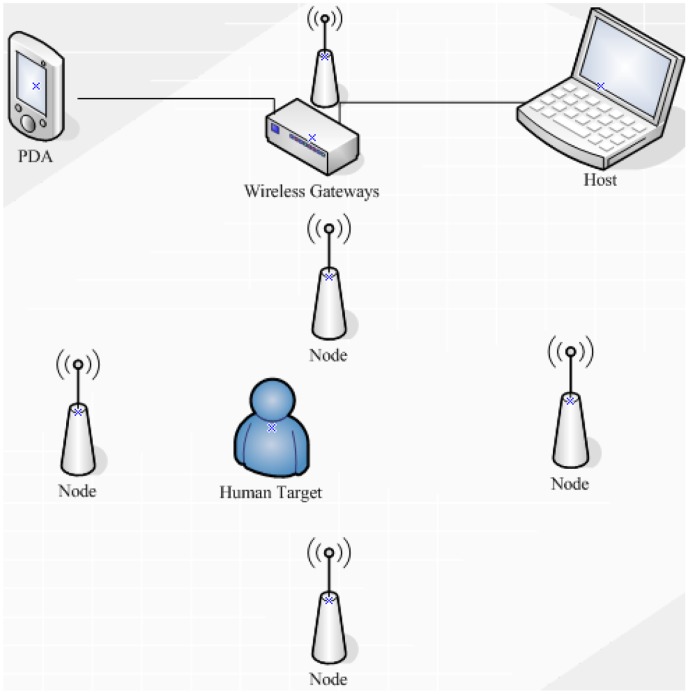
Setup of the distributed wireless pyroelectric infrared sensor system.

The major purpose of our study is to establish an effective distributed wireless pyroelectric infrared sensor network system in a specific environment. Referring to the work of Hao *et al.* [4], our system, which is shown in Figure 1, consists of four sensor MASKs (as mask sensor module), a wireless gateway and a host computer. Each MASK consists of sixteen pyroelectirc infrared sensor nodes. Each sensor node is covered by a Fresnel lens which constitutes a coded network. Once a human target moves in the network, the infrared radiation of the human target can be captured and transformed into an electrical signal which can be transmitted to the host computer to be further processed through the wireless gateway after preliminary noise reduction and data compression.

However, there are several crucial problems, as follows, need much attention before using a distributed wireless pyroelectric infrared sensor network in the process of identification of moving human targets:

(1) The effective coding of the field of view (FOV) of the sensor network in order to capture the infrared radiation of moving human targets for implementation of the subsequent algorithm.
(2) The effective data fused by multiple channel signals which are collected from four sensor MASKs.
(3) Choosing the appropriate type and number of eigenvalues to establish a target identification model.
(4) Choosing an appropriate classifier and matching the eigenvalues of human targets to the model template.

In this paper, we propose a novel way that extracts the instantaneous amplitude and instantaneous frequency of moving human target as eigenvalues by using empirical mode decomposition (EMD) and Hilbert-Huang transform (HHT) algorithms on the basis of an established distributed wireless pyroelectric infrared sensor network. After dimensional reduction, an identification model using a support vector machine (SVM) classifier was developed by training selected eigenvalues. Then, human targets can be identified by matching their subsequent captured signals to the previous model.

The rest of this paper is organized as follows: Section 2 reviews related works. Section 3 describes the distributed wireless pyroelectric infrared sensor network and system deployment. Section 4 shows the problem and proposed approaches. Section 5 shows experimental results and discussion. Section 6 concludes the paper.

## 2. Related Works

Although research about identification of human targets by applying pyroelectric infrared sensor is insufficient, many meaningful achievements have been obtained recently. In [14], Hao presented a kind of wireless pyroelectric infrared sensor tracking system composed of three modules, namely: sensor modules, synchronization and error filtering module and data fusion module. The pyroelectric infrared sensor module in this system has the ability to detect the angular displacement of moving human targets in the network. Combined with an adjustable FOV, which is realized by the Fresnel lens array, a single human body can be tracked successfully in the system. In addition, the paper also presents the problem of processing balance between light and electrical signal, the distribution of calculating load among the three types of module, and the tradeoff between performance and cost. In [15], Fang presented a real time human target recognition system using a pyroelectric infrared sensor array and the hidden Markov chain model (HMM). A series of digital sequences, which represented the characteristics of moving human targets, were generated from an array of pyroelectric infrared sensors covered by Fresnal lenses. While the statistical characteristics of moving human targets were modeled by training the HMM using the expectation maximization algorithm (EM), ultimately, the identifications of the human targets were completed by evaluating the characteristics of new data sequences and the trained HMM model under the maximum likelihood rule. Each human target, in addition, could be regarded as a distributed infrared heat source. Therefore, some special movement habits of an individual can be translated into a kind of unique and statistical label in signal space through reasonable sampling to the infrared domain. Fang, in [13], also presented a human recognition system based on pyroelectric infrared sensors and proposed an algorithm for motion feature representation of human targets by the sensor signal spectrum. The identification process of the system includes the training with different levels of velocity and the testing of various registered targets. However, the performance of this algorithm was highly dependent on the height of the sensor location and the distance to the target. Also, it can only be applied to fixed path scenarios. In [16], Zappi pointed out that the signal sensor node is usually used to trigger an alarm to judge the existence of moving targets while an array composed of many pyroelectirc infrared sensors has the capability to extract more features of moving human targets such as direction, speed and the other characteristics. Consequently, they proposed a tracking algorithm with low calculation complexity to distinguish the direction and the number of moving human targets. However, its measurement accuracy needs to be improved. Afterward, Zappi proposed in [9] another algorithm for sensor data fusion and feature extraction to track human targets moving along footpaths and higher tracing precision was obtained.

At present, there are various researches on algorithms for feature extraction and identification of moving human targets, including characterizing the appearance and extracting the features of moving human targets by building a spatial-temporal model of walking persons; extracting the features by using the image optical flow, *etc.* In fact, the features of a moving human target contain two components which are the structural component and the dynamical component. The structural component, which is a static component, is responsible for recording the human body shape information such as height and stride, while motion feature information like arm swings, body tilt and gait can vividly be represented by the dynamic component. Thus, on the basis of the two types of components, the existing algorithms can be roughly divided into two categories: the methods based on statistics (Model-Free) and the methods based on models (Model-Based).

The methods based on statistics are currently popular recognition algorithms which have been widely applied to identify moving human targets. These methods mainly focus on calculating some kinetic parameters of a human target from a continuous signal sequence and further completing classification and recognition. Due to the high dimensionality of feature data, some dimensionality reduction techniques are needed in practical applications. Niyogi proposed a theory that the head and feet of a human body have different locomotion patterns in space-time dimensionality so that some edge features of moving human targets can be extracted through handling these patterns [17]. Moreover, a kind of generalized symmetry operator was introduced by Hayfron-Acquah [18]. In order to identify moving objects, Sarkar proposed a kind of baseline algorithm in allusion to gait recognition of moving human targets [19]. In [20], little extracted the gait features based on a video system and calculated the FED vector between the sample set and a complete gait sequence of each frame image, and then the moving target could ultimately be recognized by using the HMM model. Huang, in [21], presented an algorithm to merge the motion information in both space and time domains into extension gait features by increasing regularity analysis. In addition, principal component analysis (PCA) was applied to reduce the data dimensions. Overall, due to the independence of the body structure and motion characteristics of human targets, those methods based on statistics can reduce computational complexity, which is suitable for real-time applications.

The methods based on models refer to the establishment of some model according to the specific structural characteristics of the bodies of different human targets. Therefore, different structures and mode of motion determine various established models. The classification and recognition of human targets is mainly achieved by comparing the sequence signal to the acquired model. Lee proposed the eccentricity parameters of seven interconnected ellipses to model the structure of moving human targets [22]. Moreover, Nash fitted the human body by a kind of herringbone model [23]. In [24], Wang greatly improved the recognition effect by combining the dynamic characteristics and static characteristics which usually represent the shape of a human body.

## 3. Sensor Modules and Deployment

In order to design the wireless network platform based on pyroelectric infrared (PIR) sensors, we study the design method for various video sensor platforms [25–27]. Referring to the system which was set up by Hao in [4], we built a distributed pyroeletric infrared sensor network with several sensor nodes, a wireless gateway and a host computer to detect and identify moving human targets.

### 3.1. Sensor Node

**Figure 2.**
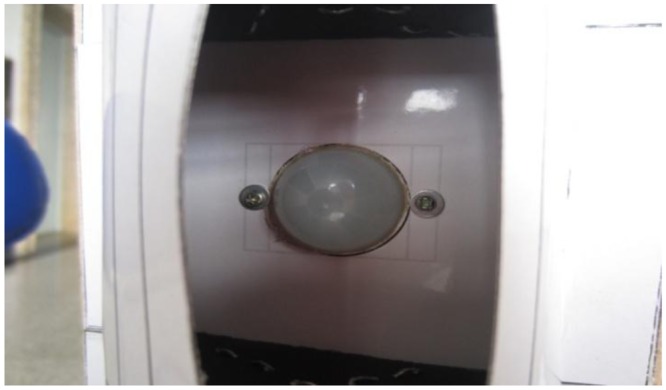
A pyroelectric infrared (PIR) sensor node covered by a Fresnel lens.

With relatively stable performance, the LiTaO_3_ film pyroelectric infrared sensor is chosen as the detection node in the system. Due to the lower receiving sensitivity of the sensor itself, every signal sensor node is covered by a Fresnel lens as shown in Figure 2. It can not only focus the infrared heat on the sensor node, but also can increase the angle and detectable distance. This was proved in some experiments in which the effective detection range could be increased from 2 m to 12–14 m. Based on the parameters and characteristics of the D205B sensor, we design the sensor node with the parameters listed in Table 1.

**Figure 3.**
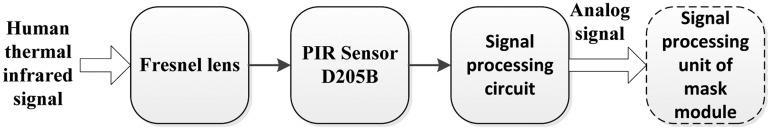
Human thermal infrared signal processing in sensor node.

As shown in Figure 3, the original thermal infrared signal of human target can be translated into an analog signal through three steps. Firstly, the original human thermal infrared signal will be focused on the pyroelectric sensor by using the Fresnel lens. Then, the infrared signal is translated into a weak electrical signal by the PIR sensor. Finally, we can get the analog signal after amplifying and filtering the weak electrical signal.

### 3.2. MASK Module

**Figure 4.**
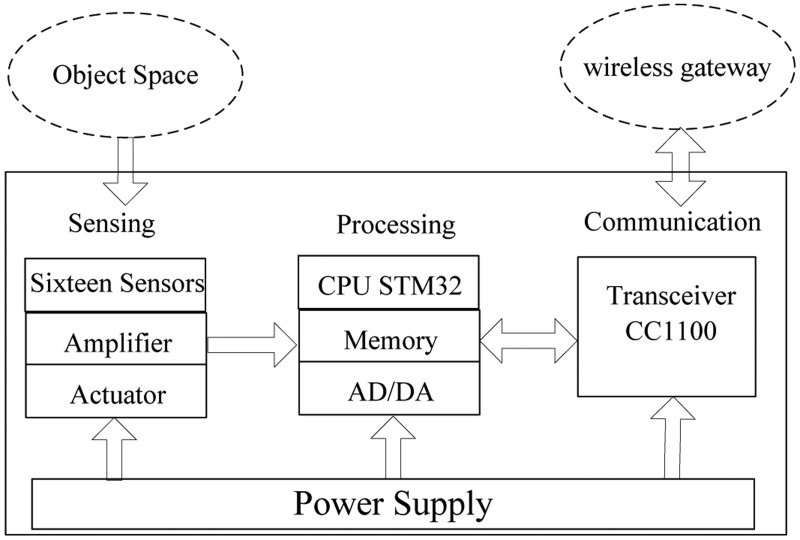
The composition module of one sensor MASK.

**Figure 5.**
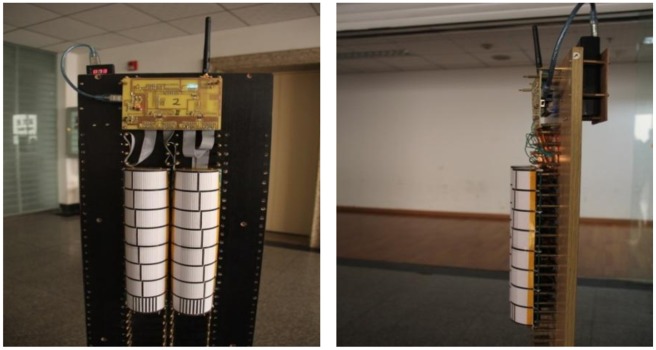
Mask sensor module.

The mask sensor module, which is shown in Figure 4, consists of a sensing unit, a processing unit, and a communication subsystem. The sensing unit is usually composed of a PIR sensor, amplifier and actuator. The original analog signal which is captured by the sensor node is first amplified by the amplifier, and then it is converted into digital signal by the analog-to-digital converter (ADC) module in the processing unit which is equipped with the STM32 CPU which has embe*d*ded 256 kB of flash memory and 48 kB of RAM for programs and data. All the sensing coordination and communication tasks are executed by this 32-bit CPU at 72 MHz. The memory subunit can store sensing data for a period of time. A communication subsystem interfaces the device to the network, and is composed of a transceiver subunit and processing circuit. The processed signal is sent to the wireless gateway by the communication subsystem on the basis of the Zigbee protocol in the specified interval. Moreover, the whole system is powered by a power unit that may be supported by an energy scavenging unit such as solar cells. The physical device is shown in Figure 5.

### 3.3. Gateway Module

**Figure 6.**
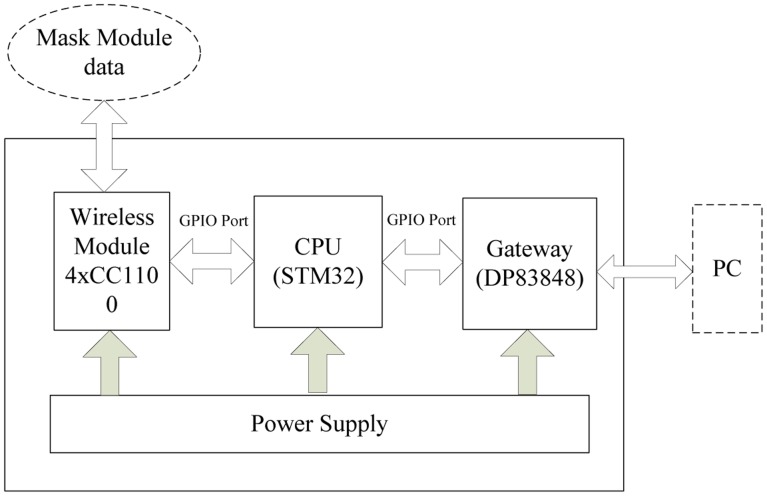
The module composition of the wireless gateway.

**Figure 7.**
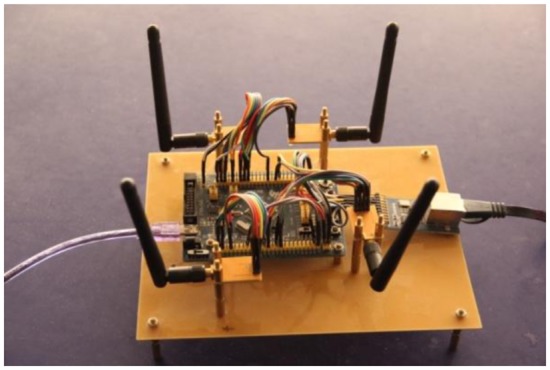
The wireless gateway module.

The gateway module is composed by four wireless module units, STM32 processor and gateway unit. The human target signals which are detected by each MASK, are all transmitted to the four CC1100 wireless modules in the specified interval. Since the four wireless modules have four kinds of different channels and connect with the STM32 processor by General Purpose Input/Output (GPIO), the STM32 processor can independently process the different data streams from the MASK modules, and it can also complete wireless data acquisition at the same time. Moreover, sensed data will be transmitted to the host computer through an Ethernet interface for further processing and display. The whole structure and a photo of the wireless gateway module are shown in Figure 6 and Figure 7, respectively. The whole system is also powered by a power unit.

### 3.4. Location of Human Targets

In order to accurately extract the features of human targets moving in the network, we locate moving human targets relying on the feedback from various coded signal of sensor nodes that detect the entire network FOV which has been segmented and coded. On account of the fact that the pyroelectric infrared sensor is sensitive to certain human body radiation wavelengths, different the sensor nodes of the system can capture the heat radiation and translate it into a time-varying signal when a human target moves in the detection area [28]. This is achieved in the following ways:

(1) Distributing the sixteen sensor nodes of each MASK as a fan, which can make the detection area cover a certain range. In order to improve precision, the detection area is coded and overlap of the visible range between adjacent sensor nodes is allowed.
(2) Distributing the calculation workload between the MASK, the wireless gateway and the host computer. We build the system according to the process of signal filtering, threshold value judgment, error detection and path tracking.
(3) Balancing the performance and cost of the system and using fast Fourier transform (FFT) as the filtering algorithm to process signals.

**Figure 8.**
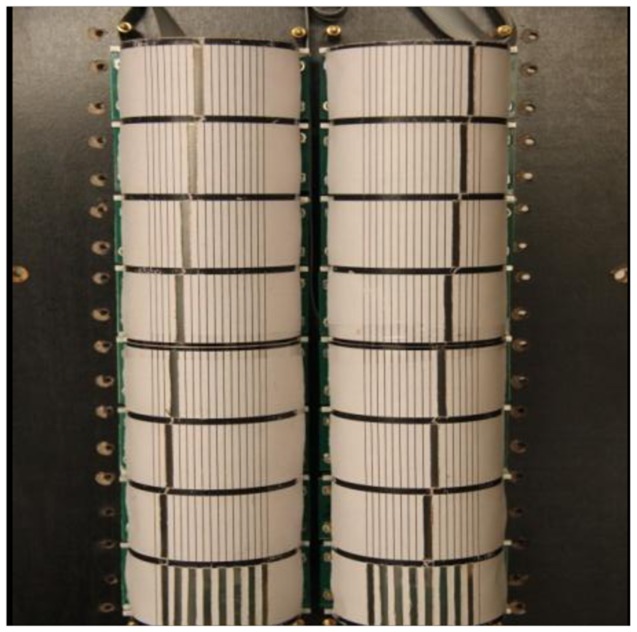
Coded mask sensor module.

To facilitate location of human targets, the MASK in the system is designed as two-column and consists of sixteen sensor nodes. Two-column nodes give two separable detection ranges of 70°. Each test scope contains seven different perceptual visibility detection areas. Thus, the average angular resolution of sensor MASK module is 10°. Its structure is shown in Figure 8. This kind of visible design also makes it convenient to realize data association with human targets.

**Figure 9.**
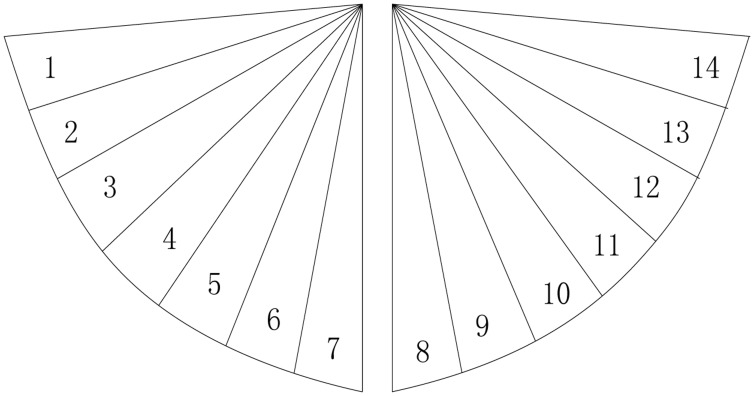
Division of the detection area of the system.

**Figure 10.**
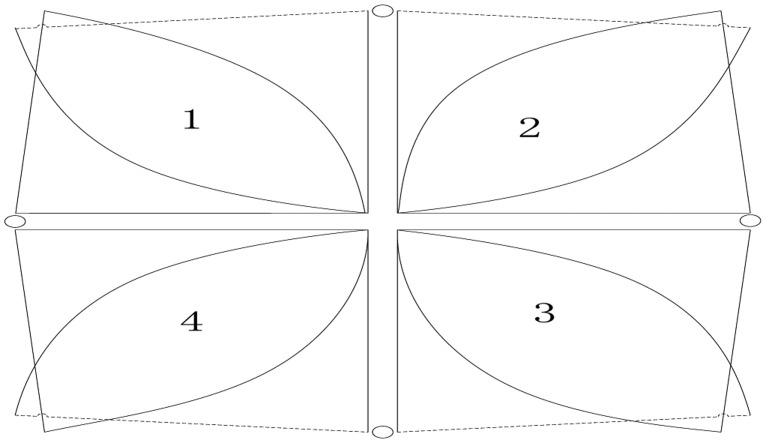
Field of view (FOV) of the whole sensor network.

Figure 9 shows the division of the detection area of the system. The outward fan area is divided into 14 sub areas which are numbered 1 to 14, respectively. The sensor nodes of each MASK are also numbered 1 to 14; moreover, each sensor node has a corresponding binary value of 1 with output and 0 without output. Because the target crosses different numbered areas, different binaries will be obtained when a human target moves in the sensor network. We can simply locate the human target by simply associating the number with the actual physical coordinates. However, this method can be achieved only for different directions of one fan area. In order to locate the human target in the whole sensor network, we try to apply the collaboration of multiple MASKs to obtain more precise location information. As shown in Figure 10, there are some overlapping areas, which can be numbered 1 to 4, at the adjacent detection range of MASKs. Hence, the human target can be detected by two sensor MASKs simultaneously when it moves in those adjacent ranges, so more precise coordinate information can be calculated according to the relation between the locations of two MASK sensors themselves and the angle of the detection area.

## 4. Problem and Proposed Approaches

In this section, we present the multiple human targets identification problem in terms of a sensor observation model. The process of this problem can be formulated as five parts: data fusion, feature extraction, data learning, hypothesis testing and final identification.

### 4.1. Empirical Mode Decomposition

The idea of the empirical mode decomposition (EMD) algorithm was first proposed and developed by Huang and coworkers [29]. This algorithm is a self-adaptive signal processing method, which is very suitable for application in non-stationary signal processing scenarios. By decomposing the data first into some intrinsic mode functions (IMFs), it can solve those problems involving non-stationary data that the Hilbert transform cannot be used on. An IMF represents a kind of oscillatory mode embedded in the signal and every single IMF should satisfy two conditions: firstly, the number of extreme and zero-crossing must either be equal or differ at most by only one among the whole dataset; secondly, the mean value of the envelope is defined by the local maxima and the local minima must be zero at any point in dataset. The processing of a signal *X*(*t*) using the EMD algorithm can be represented as follows:

Initialize *r_i_*(*t*) = *X*(*t*), *i* = 1;
Initialize *h_ik_*(*t*) = *r_i_*(*t*),*k* = 1;

Calculate the upper and lower envelopes of *h_ik_*(*t*) by cubic spline interpolation in its local maxima and minima.

Calculate the mean value *m_ik_*(*t*) of the upper envelop and lower envelop, then let. *h_i_*_(_*_k_*_+1)_(*t*)=*h_ik_*(*t*)-*m_ik_*(*t*).

If *h_i_*_(_*_k_*_+1)_(*t*) satisfies the two conditions and becomes an IMF, then set *c_i_*(*t*)=*h_i_*_(_*_k_*_+1)_(*t*). Else let *k*=*k*+1 and repeat the steps above.

Calculate the residue *r_i_*_+1_(*t*) = *r_i_*(*t*)−*c_i_*(*t*). If *r_i_*_+1_(*t*) contains at least two extreme, it is treated as input to derive the next IMF.

Else the process is finished and *r_i_*_+1_(*t*) is denoted as the final residue *r_f_*.

Ultimately, the original signal *X*(*t*) can be decomposed into a set of IMFs *c_i_*(*t*) and a residue *r_f_* as:

(1) $$X(t)=\sum_{i=1}^{j}c_{i}(t)+r_{f}$$

IMFs are determined by the signal itself rather than pre-determined functions so the EMD algorithm is a self-adaptive signal process.

### 4.2. Acquisition and Preliminary Processing of Signal

Generally, there are three data fusion methods for the signal which was acquired from multiple sensors of the same type working together. Namely, sampling fusion, feature fusion and decision fusion.

Theoretically, when one or multiple human targets move in the detection areas, for *N* MASKs, each having *m* pyroelectric infrared sensor nodes, the ensemble data of the system can be denoted as **D**=[**d**_1_*^T^*…**d***_N_^T^*]*^T^*, where d*_i_*=[*d_i_*_1_…*d_iL_*] is an *m* × *L* event sequence. The feature sequence selection is to choose *M* sequences containing information about motion human targets and then denoting as ξ(·). In addition, for fusing the signal characteristics, the highest frequency of the signal sequences of each MASK will be selected as the feature sequence.

For the fusion strategy, the probability of a joint feature vector is in accord with a joint hypothesis of the human target. Thus, we can refer to the Formula (2) as the fusion decision rule:

(2) $$X\in \left\{ \begin{array}{l} H_{0},\text{if}\max_{i}p(X|H_{i})<\gamma \\ H_{i},i=\text{arg}\max_{i}p(X|H_{i}),\text{otherwise} \end{array}\right.$$

**Figure 11.**
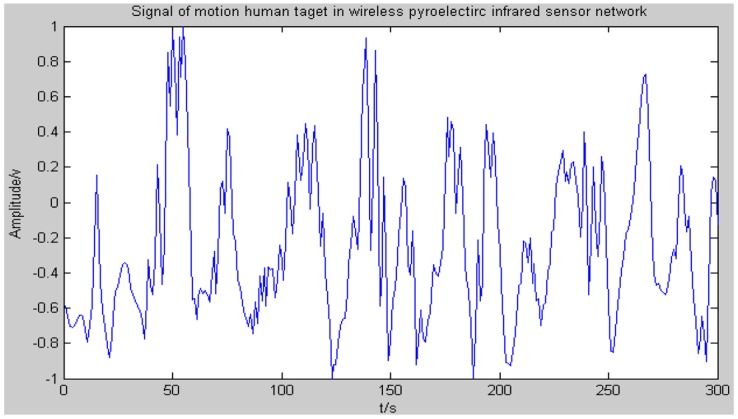
The signal of a moving human target in a wireless pyroelectric infrared sensor network.

The binary decision vector $\left[D_{k}^{1}\ldots D_{k}^{N}\right]$ can be obtained for the *k*-ith human target, and then the final decision can be completed by using the majority voting strategy. Figure 11 shows the signal which is finally transferred to the host computer.

### 4.3. Time-Frequency Analysis for the Signal of Moving Human Target

**Figure 12.**
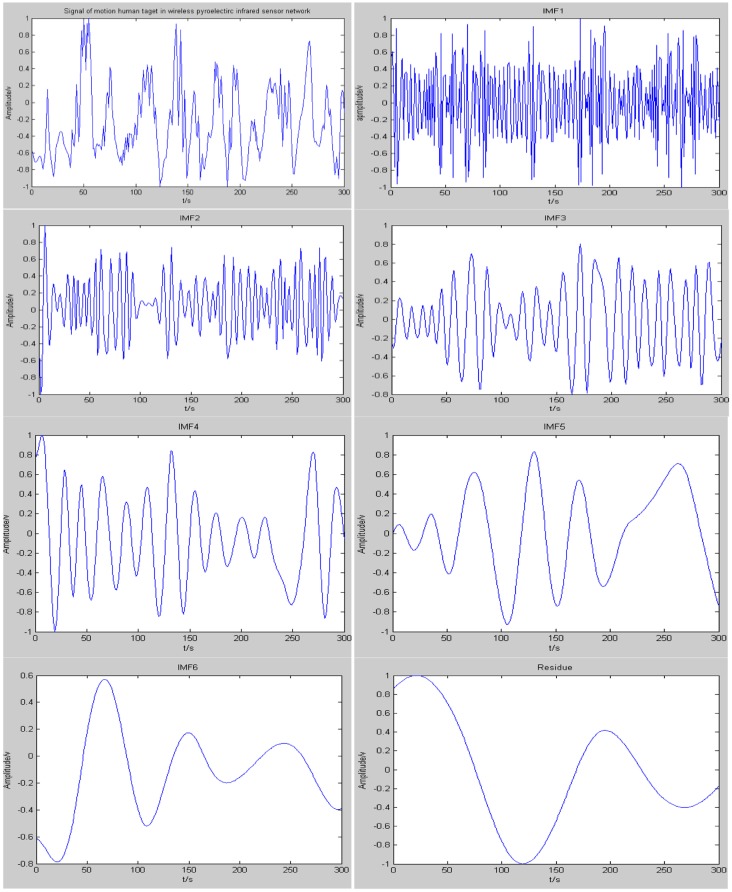
The original signal of a moving human target and its intrinsic mode functions (IMFs) using the empirical mode decomposition (EMD) algorithm.

Compared with other algorithms, EMD has the capability to demonstrate the changing features of a signal in different frequency ranges based on adaptively decomposing the time domain non-stationary signal. The decomposition results of a moving human target signal which is captured by a pyroelectirc infrared sensor are shown in Figure 11. It can be clearly seen from Figure 12 that the original signal is decomposed into six IMF components and one residue after several cycles of iterative calculations using the EMD algorithm. Each IMF component represents the instantaneous amplitude features of the original signal in a different frequency range. Consequently, appropriate IMF components of the frequency range can be selected to compose feature values of specific moving human targets.

For analysis of the signals of a human target moving in a pyroelectirc infrared sensor network, signal decomposition in the time domain often cannot accurately demonstrate the movement features of various human targets due to the characteristics of the signal itself. Therefore, transforming the signal to the frequency domain for further decomposition and analysis is also needed in addition to the time domain signals. Pertinent literature has proved that the signal from a pyroelectric infrared sensor node is usually a non-stationary one whose obvious feature is the existence of time-varying frequency eigenvalues.

The traditional Fourier transform, is the classical physics tool for periodic signals. Its essence is the general feature within the whole frequency domain range and it is unable to show the variation of instantaneous frequencies (IF) of the signal. Thus, Huang proposed the Hilbert-Huang transform (HHT) on the basis of the EMD algorithm. The basic idea is that the original time signal is decomposed into a combination of numbers of IMFs by EMD. Then, some meaningful instantaneous amplitudes and instantaneous frequencies are calculated by using the Hilbert transform for every IMF component, so that the Hilbert spectrum with time-varying information will be obtained [30].

**Figure 13.**
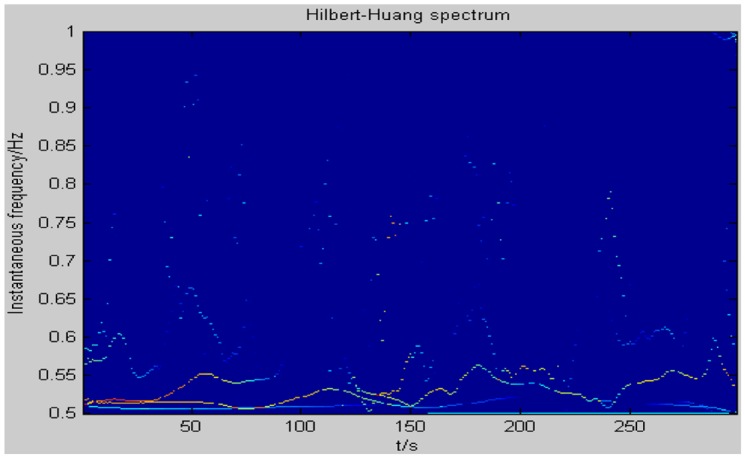
The two-dimensional (2D) image of instantaneous frequencies.

Figure 13 shows the two dimensional distribution of instantaneous frequencies of a moving human target signal. It can be clearly seen from Figure 12 that the energy of the human target is mainly concentrated in the vicinity of 0.5 Hz during 0 to 300 s. When the distance between the human target and the MASK is 7.5 to 8 m, which is the best detection distance for a pyroelectric infrared sensor, the instantaneous frequencies are just concentrated on 0.5 Hz corresponding to the velocity of the moving human target in the network, which is about 1 m/s. In addition, that is a reasonable normal walking velocity.

### 4.4. Classifier

Currently, the neural network method is usually applied in pattern recognition applications and data classification. Its primary idea can be simply described as a hyperplane generated and moved randomly by the system until the centralized data, which belong to inhomogeneous points, is located on different sides of the sub-hyperplane. This random processing mechanism determines the final classified hyperplane will be quite close to the points in the training set. Hence, it will not be the optimal solution in most cases. In order to overcome the defects of the neural network, we consider seeking a special data classification hyperplane which makes the training points' distance to the optimal hyperplane as far as possible, so as to achieve the purpose of data point optimization classification, and then a new general learning machine, support vector machine, emerges [31]. Modeled by the principle of structural risk minimization, the support vector machine is expected to minimize risk effectively and significantly improve the recognition ability of its model. In addition, it shows many unique advantages in resolving pattern recognition and classification problems of small samples, nonlinear and high dimensional data. It can be popularized to learning problems of other machines such as function fitting and so on. Theoretically, a support vector machine can realize optimal classification of linear data while Vapnik mapped data into high dimensional space using some kinds of kernel function. However, in the process of mapping from the low dimensional feature space to the high optimal hyperplane, it cannot be calculated directly in most cases because of the speedy increase of the space dimensions. By defining various kernel functions, a decision function is obtained as follows:

(3) $$f(x)=\text{Sgn}\left\{\sum_{i=1}^{n}y_{i}\alpha_{i}^{*}K(x_{i}\cdot x)+b^{*}\right\}$$

As a parameter symbol function, the results of this decision function are only two options which either is +1 or −1. According to Equation (3), it is known that the final discriminant function actually contains the inner product and summation of support vectors, thus the complexity of the recognition algorithm ultimately depends on the number of support vectors. Through the analysis above, it is known that the data classification in support vector machines is mainly completed by a kernel function, thus it is proved that different support vector machine classification algorithms will appear for different selections of kernel functions. Currently there are three kinds of mainstream kernel function:

(1) Linear Kernel defined as *K*(*x*,*x_i_*) = *x.x_i_*.
(2) Polynomial Kernel defined as *K*(*x*,*x_i_*) = [(*x.x_i_*+1)]*^q^*. And the support vector machine with this kernel function is a *q*-order polynomial classifier.
(3) Radial Basis Function Kernel defined as *K*(*x*,*x_i_*) = exp{-|*x*-*x_i_*|/2σ^2^}. The support vector machine with this kernel function is a radial basis function classifier. In addition, every center of a base function corresponds to a support vector and the every weight vector of output is allocated by the algorithm automatically.

In allusion to the two support vector machine classification problems, namely that it cannot be directly applied in linearly separable multiclass pattern recognition such as classification of multiple human targets because it was first proposed for two categories. To settle this problem, a support vector machine classifier can be constructed for each human target. Each classifier of each human target is responsible for the distinction between the detected points which belong to this human target and the points belonging to other targets. The final result is decided by the support vector machine classifier whose output is the most far away from the interface *w*·*x*+*b*.

## 5. Experiments and Results

**Figure 14.**
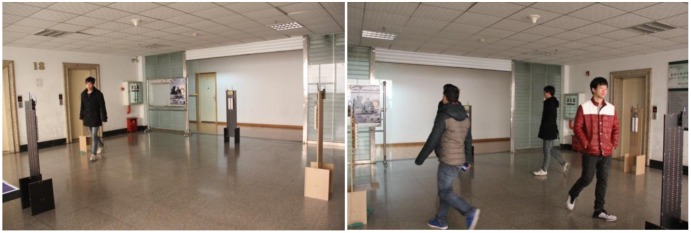
The experiment scene and a moving human target.

The experimental distributed wireless pyroelectric infrared sensor network has been implemented in a 10 m × 10 m room without any obstacles. One or more human targets were moving in the detection area. Figure 14 shows the experiment scene.

### 5.1. Human Target Tracking and Locating

**Figure 15.**
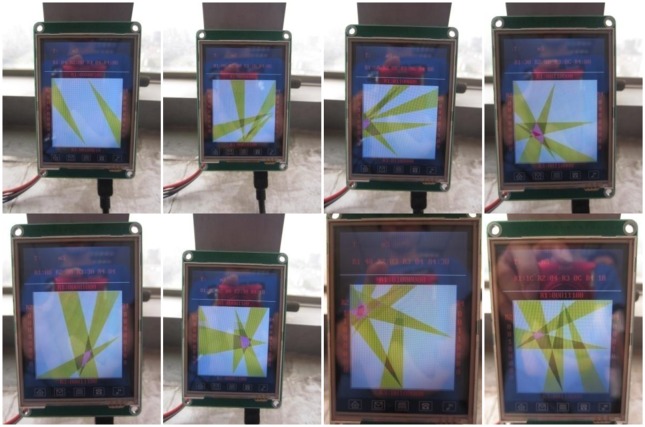
Snapshots of tracked targets shown on a personal digital assistant (PDA).

When one or more human targets move in the detection area, the host computer can process the signals received from the wireless gateway module and the tracking results are displayed on the color screen of a personal digital assistant (PDA). It uses the graphical display to indicate the relative position of the human targets in real-time. Figure 14 displays several snapshots of targets tracked using the PDA. It is clearly shown in Figure 15 that the purple overlapping region is the area which can be detected by three MASKs and the red overlapping region is the area which can be detected simultaneously by all four MASKs. Compared with the actual position of the moving human target, this PDA system can track and locate the moving human targets within a certain error range.

### 5.2. Recognition of Human Targets

For human target identification, we used four pyroelectric infrared sensor MASKs which have been described in Section 3. The human targets randomly walked at the same time at a normal rate of speed inside the detection area that is illustrated in Figure 14. The human target signals were received and fused by four MASKs; next, the signals were transmitted to the wireless gateway module, which is illustrated in Figure 7, and sent by the upper machine to a host computer for subsequent processing.

**Figure 16.**
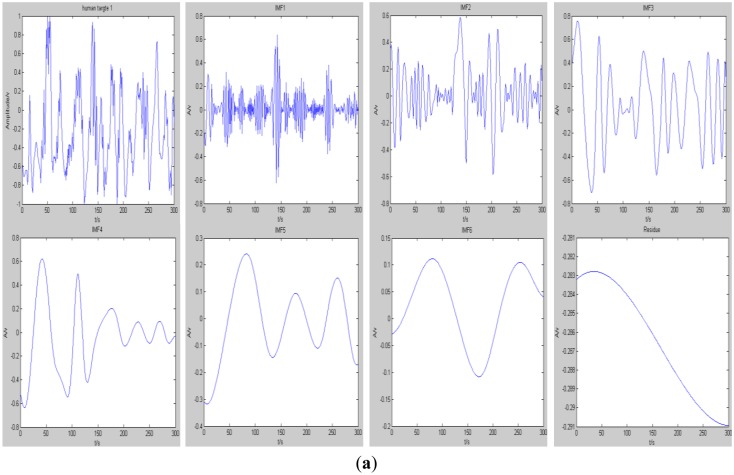

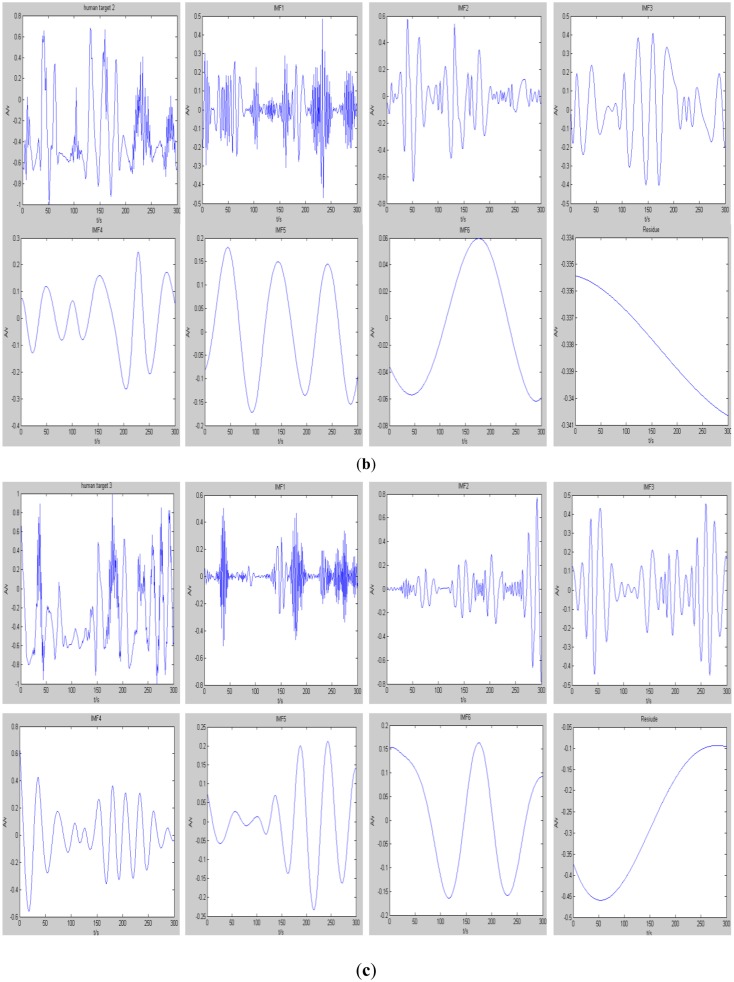
The signal and the IMFs corresponding to three human targets: (**a**) the first human target; (**b**) the second human target; and (**c**) the third human target.

Figure 16a–c display the signals of three different moving human targets and the IMFs of the corresponding human target signals were decomposed by the EMD algorithm. For the three human targets, we use principle component analysis (PCA) to extract their features from the combination of seven IMFs of each human target. Then, a SVM classifier is trained and modeled by those extracted features and at last three human targets can be identified by using the trained SVM classifier in three different ways.

**Figure 17.**
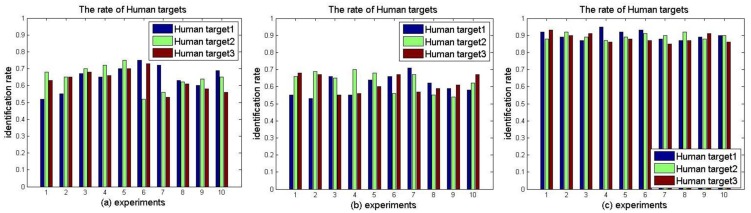
The rate of identification of human targets: (**a**) the identification rate using features extracted from all IMFs; (**b**) the identification rate using features extracted from four IMFs; and (**c**) the identification rate using features combined with IMFs and instant frequencies.

(a) Extracting the features from the data matrix which is combined with all IMFs of each human target. Figure 17a displays the identification rate of three human targets using the features which were extracted from all IMFs. The identification rates of repeated ten experiments of three targets are all around 70%.
(b) Extracting the features from the data matrix which is combined with IMF1, IMF2, IMF3, and IMF4 of each human target. Figure 17b illustrates the identification rate of three human targets using the features which were extracted from combination of IMF1, IMF2, IMF3, and IMF4 of three targets. The identification rates of repeated ten experiments of three targets are also all around 70%.
(c) For the reason that the most time domain features are contained in the components of IMF1, IMF2, IMF3, IMF4, the identification rates in experiment (a) are basically as same as in experiment (b), for the reason that the most time domain features are generally contained in the components of IMF1, IMF2, IMF3, and IMF4. However, the identification rates are relatively low if we use the features merely extracted from time domain IMFs components. Therefore, in experiment (c) we chose the combined features of IMF1, IMF2, IMF3, IMF4 and instant frequencies transformed by HHT as eigenvectors to recognize the three human targets. The identification rates, which are shown in Figure 17(c), basically stabilize around 90% due to the fact those eigenvectors already include most features both in time domain and frequency domain.

## 6. Conclusions

In this paper, we present a wireless distributed pyroelectric infrared sensor network system and a novel feature extracting method using an EMD algorithm for tracking and identifying moving human targets. The system consists of four MASK modules, a wireless gateway module, PDA and a host computer. Moreover, compared to other means of signal feature extraction, the method we applied in this paper is the EMD and HHT algorithms which can extract instant features in either the time domain or frequency domain. In addition, we use a SVM to construct a classifier to identify multiple human targets. The results of repeated experiments show that the proposed sensor system and EMD and HHT algorithms can work as a reliable biometric system for a few human targets.

## Figures and Tables

**Table 1.** The parameters and characteristics of the D205B sensor.

**Parameters**	**Value**
IR Receiving Electrode	0.7 × 2.4 mm, 4 elements
Sensitivity	≥4300 V/W
Detectivity (D*)	1.6 × 108 cm·Hz^1/2^/W
Supply Voltage	3–15 V
Operating Temperature	−30–70 °C
Offset Voltage	0.3–1.2 V
FOV	150°
